# Immunochemotherapy for small cell lung cancer with paraneoplastic Cushing syndrome: A case report and literature review

**DOI:** 10.1097/MD.0000000000041036

**Published:** 2024-12-20

**Authors:** Ling Yu, Yanlong Li, Caiyu Li, Xiangjun Qi, Yeding Lin, Yuanliang Li, Hanrui Chen, Lizhu Lin

**Affiliations:** a The First Affiliated Hospital of Guangzhou University of Chinese Medicine, Guangzhou, China; b The First Clinical School of Guangzhou University of Chinese Medicine, Guangzhou, China.

**Keywords:** case report, Cushing syndrome, durvalumab, immunochemotherapy, paraneoplastic, serplulimab, small cell lung cancer

## Abstract

**Rationale:**

Paraneoplastic Cushing syndrome (PCS) is an adverse prognostic factor for small cell lung cancer (SCLC) patients. Retrospective studies have shown that the median survival of SCLC complicated with PCS was <7 months. No immunochemotherapy has been recorded in the treatment of SCLC with PCS. Previous preclinical and clinical studies have suggested glucocorticoid exposure may affect the efficacy of immunotherapy.

**Patient concerns and diagnosis:**

A 60-year-old man was admitted for his irritability and palpitation. During hospitalization, a chest computed tomography scan revealed a lobar soft tissue shadow in his left lower lung. He was diagnosed as limited-stage SCLC (T2bN1M0 IIB) with PCS, ultimately.

**Interventions and outcome:**

The patient received 4 courses of immunochemotherapy of etoposide plus platinum with durvalumab and 1 adjuvant radiotherapy alone in 2022 for his limited-stage SCLC, and underwent 5 courses of immunochemotherapy of irinotecan plus platinum with serplulimab in 2023 for his extensive-stage SCLC. The patient achieved a long survival of 20 months.

**Lessons:**

The case preliminarily demonstrated the efficacy of immunochemotherapy in the management of SCLC complicated with PCS. The regime of serplulimab with irinotecan-based chemotherapy also indicated its satisfactory efficacy as a second-line treatment for extensive-stage SCLC. Furthermore, the case has highlighted that the management of hypercortisolism, the improvement of myelosuppression, and the prophylaxis against infection were 3 hinges for the continuation of immunochemotherapy and the holistic management of SCLC with PCS.

## 1. Introduction

Small cell lung cancer (SCLC) accounts for only 14% of all lung cancer cases but exhibits an extremely lethal capability due to its rapid proliferation, early metastasis, and poor prognosis, with a 5-year survival rate of <7%.^[1,2]^ SCLC is further complicated by its neuroendocrine nature. Paraneoplastic Cushing syndrome (PCS), present in approximately 5% of all SCLC cases, is particularly associated with an even worse prognosis, characterized by decreased sensitivity to chemotherapy and increased complications, especially metabolic disorders and opportunistic infections.^[3,4]^ An early study identified PCS as an adverse prognostic factor as that patients with PCS more likely to experience premature death than those without.^[5]^ Another latest research suggested that PCS was characterized by extensive tumors and a diminished response to both first-line and second-line chemotherapy.^[6]^ Multiple retrospective studies have shown that the median survival of SCLC with PCS patients was <7 months.^[5–10]^ However, none of the antitumor regimens recorded in these reports mentioned immunochemotherapy.^[11]^

Before the introduction of immunotherapy into the therapeutic strategy of SCLC, etoposide plus platinum combination chemotherapy had been the preferred regimen for both limited and extensive-stage SCLC (ES-SCLC) for over 30 years.^[12,13]^ This also applied to the management of SCLC complicated with PCS in the past. Atezolizumab and durvalumab were approved by the USA Food and Drug Administration (FDA) on March 3, 2019 and March 30, 2020, respectively. Since then, the immunochemotherapy consisting of atezolizumab or durvalumab in combination with etoposide-platinum has been considered the new standard of care in first-line setting for SCLC.^[12,13]^ A single-arm phase II study suggested promising efficacy of durvalumab with concurrent chemoradiotherapy in limited-stage SCLC (LS-SCLC) as well as another phase 3 randomized clinical trial (ASTRUM-005) demonstrated improved overall survival with serplulimab plus chemotherapy compared to chemotherapy alone in ES-SCLC.^[14,15]^ However, no evidence has been reported on the backline use of serplulimab for the treatment of SCLC.

On the one hand, previous preclinical and clinical studies have suggested glucocorticoid exposure may affect the efficacy of immunotherapy.^[16–18]^ On the other hand, as far as our knowledge is concerned, there have been no reports on the immunochemotherapy for SCLC complicated with PCS. Nevertheless, here was an SCLC with PCS patient who received immunochemotherapy with first-line durvalumab followed by second-line serplulimab for his limited and extensive disease stages, sequentially. The patient achieved a long survival of 20 months, which far exceeded results from previous studies. In the current report, the medical management for his complicated disease is presented to share some shallow experience for clinical treatment and provide some primitive ideas for further clinical study.

## 2. Case presentation

The case has been reported following the CARE guidelines.^[19,20]^

### 2.1. The diagnosis of small cell lung cancer

**Figure 1.**
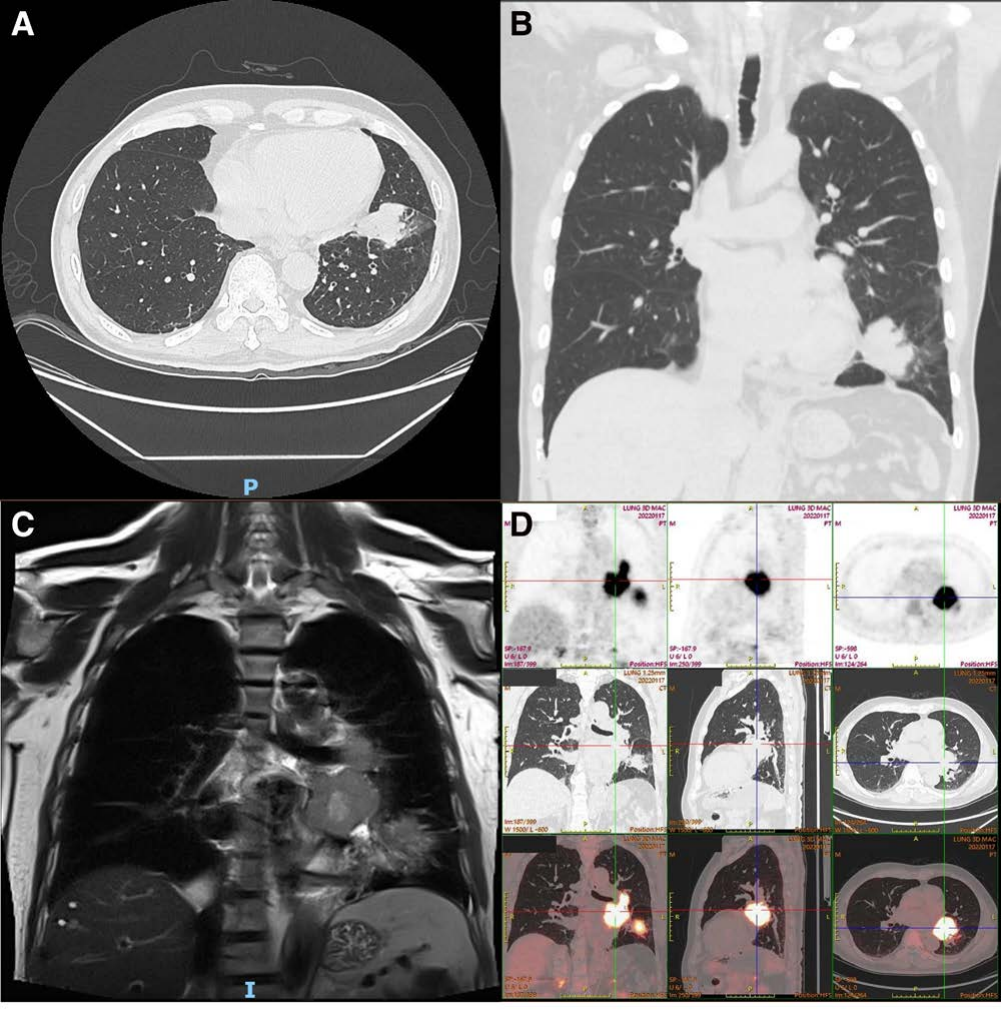
Imaging findings in thorax. (A) CT from transverse view. (B) CT from coronal view. (C) MRI from coronal view. (D) PET-CT. CT = computed tomography, MRI = magnetic resonance imaging, PET-CT = positron emission tomography-computed tomography.

**Figure 2.**
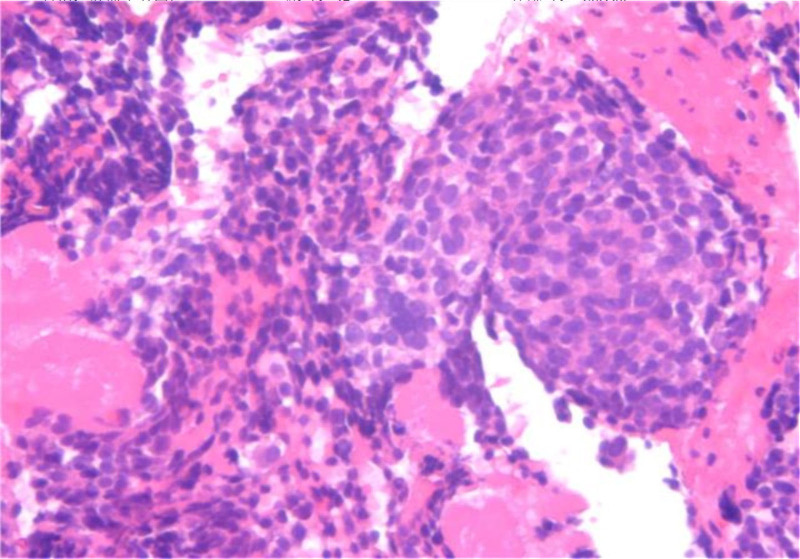
Pathology findings from pulmonary lesion.

In January 2022, a 60-year-old man who had a smoking history of more than 40 years and had quit smoking 6 years ago, came to sought medical help for his irritability and palpitation. During hospitalization, a chest computed tomography (CT) scan revealed a lobar soft tissue shadow in the left lower lung with a large cross-sectional area of approximately 44 × 28 mm and several enlarged lymph nodes in the left hilum, the largest of which was approximately 59 × 51 mm (Fig. 1A and B). In conjunction with the magnetic resonance imaging (MRI) enhancement scan, a lung cancer with metastatic lymph nodes was considered possible (Fig. 1C). Subsequently, a biopsy pathology of the tissue obtained by bronchoscopy showed an irregular nest-like infiltration of heteromorphic cells with homogenous nuclei and the immunohistochemistry exhibited: CK (+), TTF-1 (+), Napsin A (−), P40 (−), CD56 (+++), Syn (+++), CgA (+++), programmed cell death ligand 1 (−), SSTR2 (+), Ki-67 (about 60%+) (Fig. 2). The whole-body positron emission tomography-CT and brain MRI suggested no other metastases (Fig. 1D). Ultimately, the patient was diagnosed as LS-SCLC (T2bN1M0 IIB) according to the 8th edition lung cancer stage classification from American Joint Committee on Cancer, Union for International Cancer Control and International Association for the Study of Lung Cancer.^[21]^ Genetic testing indicated that programmed cell death ligand 1 was negative (tumor cell proportion score <1%, combined positive score <1), tumor mutation burden was low (5.03 Muts/Mb), and microsatellite was stable.

In addition, the patient had hypertension disease for over 20 years and diabetes mellitus for over 5 years, with regular medication usage to control his blood pressure and blood glucose.

### 2.2. Diagnosis and management of paraneoplastic Cushing syndrome

As for his irritability and palpitation, in combination with elevated TRAb (5.88 IU/L), anti-TPO (24.15 IU/mL), free T4 (17.14 pmol/L), decreased TSH (0.009 mIU/mL), and nodular goiter on thyroid ultrasound, the patient was diagnosed with hyperthyroidism. Furthermore, in consideration of the neuroendocrine activity common to SCLC and the elevated neuron-specific enolase (36.42 ng/mL), we examined the patient’s corticosteroid production. According to the results from 2 morning blood cortisol and adrenocorticotropic hormone (ACTH) (46.203 µg/dL for cortisol and 209.086 pg/mL for ACTH on the first day, >60 µg/dL for cortisol and 251.612 pg/mL for ACTH on the second day), and elevated urinary 17-hydroxycorticosteroids, he was diagnosed as SCLC with PCS, ultimately.

Hence, octreotide and mifepristone were prescribed to control the paraneoplastic neuroendocrine activity of the SCLC, and correspondingly the patient’s hyperthyroidism resolved.

### 2.3. Immunochemotherapy in 2022: etoposide plus platinum with durvalumab for limited-stage SCLC

In March, the patient received the first cycle of first-line immunochemotherapy and the specific regimen included: etoposide (160 mg, orally, days 1–3), carboplatin (500 mg, intravenously, day 1), durvalumab (1000 mg, intravenously, day 1). Following the treatment, the patient suffered a fourth-degree myelosuppression and an infectious fever with *Klebsiella pneumoniae*. However, the good news was that the CT suggested a partial response. The tumor shrank into 22 × 12 mm and the largest lymph node diminished to 11 × 14 mm. The postponed second cycle immunochemotherapy was administrated in May, the fourth-degree myelosuppression and infectious fever returned as soon as the treatment completed. In June, the treatment was interrupted again for an acute onset of chronic heart failure.

In July and August, in consideration of repeated fourth-degree myelosuppression following immunochemotherapy, the patient received a course of adjuvant radiotherapy alone and the specific dose was 60 Gy in 26 fractions. Subsequently, CT suggested the tumor shrank into approximately 6 × 4 mm. In September, the patient was caught by an infectious fever again. The next-generation sequencing of alveolar lavage fluid revealed multiple infections with *Acinetobacter baumannii, Pneumocystis jirovecii*, Torque teno virus, and Human herpesvirus type 5.

**Figure 3.**
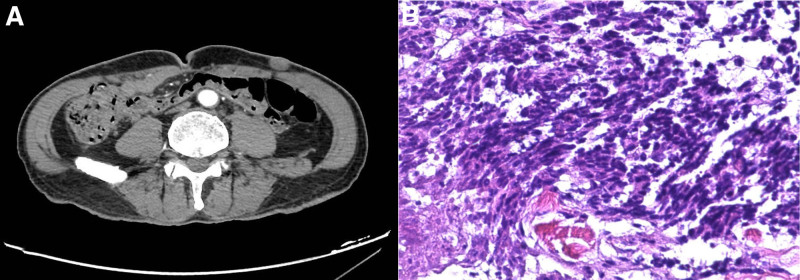
Imaging findings of hypogastrium (A) and pathology findings of the lesion in left lower abdominal wall (B).

In October, the abdominal CT and MRI suggested multiple metastases in his liver (approximately 8 mm for the largest one) and a nodule in his left lower abdominal wall (approximately 17 × 11 mm) (Fig. 3A). Then, the patient received 2 cycles immunochemotherapy without platinum: etoposide (100 mg, orally, days 1–10), durvalumab (1000 mg, intravenously, day 1). In December, the nodule in the abdominal wall was resected and pathological findings were consistent with metastatic SCLC, with focal differentiation of large cell carcinoma (Fig. 3B).

### 2.4. Immunochemotherapy in 2023: irinotecan plus platinum with serplulimab for ES-SCLC

The CT-enhanced scan of chest, abdomen, and pelvis suggested progressed lung lymph nodes with increased and enlarged liver metastases (approximately 16 mm for the largest one). In addition, an enlarged lymph node in his left inguinal region (approximately 9 mm) was newly indicated. As the SCLC had progressed to an extensive-stage, the second-line immunochemotherapy of irinotecan plus platinum with serplulimab was started in 2023.

In January and February of 2023, the patient received 2 cycles of second-line immunochemotherapy and the specific regimen included: irinotecan (110 mg, intravenously, day 1 and day 8), cisplatin (30 mg for day 1 and day 8, 20 mg for day 2 and day 9, intravenously), serplulimab (200 mg, intravenously, day 1). Subsequently, the CT suggested a partial response. The largest liver metastasis shrank into approximately 8 mm with lung lymph nodes diminished. But an uneven bone density area in his left iliac crest was newly reported, which couldn’t rule out the possibility of metastasis. In March, the patient completed his third cycle immunochemotherapy: irinotecan (110 mg, intravenously, day 1 and day 8), carboplatin (200 mg for day 1, 190 mg for day 8, intravenously), serplulimab (200 mg, intravenously, day 1). And trilaciclib was administrated before immunochemotherapy to protect bone marrow stem cells. Unfortunately, the patient suffered myelosuppression, infectious fever, acute heart failure, again.

In May and August, the patient underwent his fourth and fifth cycles immunochemotherapy: irinotecan (110 mg, intravenously, day 1 and day 8), cisplatin (40 mg, intravenously, day 1 and day 8), serplulimab (200 mg, intravenously, day 1), cisplatin was adjusted back and dosed down for his severe myelosuppression. And trilaciclib was routinely administrated before immunochemotherapy. In the interim 2 months, due to a significant pleural effusion, the patient received a separate regimen of anlotinib (12 mg, orally, days 1–14) + bevacizumab (400 mg, intrapleurally, every 3 weeks).

However, the CT in July suggested a suspicious metastasis in the fourth lumbar vertebra and an atrial septal aneurysm with a patent foramen ovale. On the CT performed on September 9, the largest liver metastasis had grown into 71 × 61 mm, the lymph node in his left inguinal region had enlarged into 30 × 17 mm, and the bone metastasis in his left ilium exhibited further progression with a soft tissue mass developed.

Ultimately, on September 23 in 2023, the patient succumbed to respiratory defunction and cardiac failure due to a severe infection following myelosuppression.

**Figure 4.**
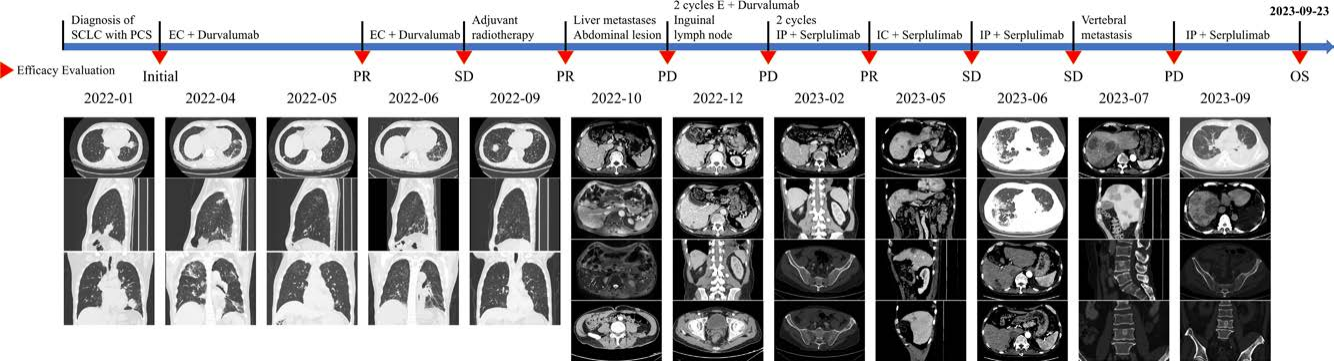
Treatment timeline and efficacy evaluation. PD = progressive disease, PR = partial response, SD = stable disease.

In total, during his 20-month-long survival, the patient received 4 courses of immunochemotherapy of etoposide plus platinum with durvalumab (the last 2 courses excluded platinum) and 1 adjuvant radiotherapy alone in 2022 for his LS-SCLC; subsequently, he received 5 courses of immunochemotherapy of irinotecan plus platinum with serplulimab in 2023 for his ES-SCLC. The treatment timeline and efficacy evaluation are presented in Figure 4.

## 3. Discussion

### 3.1. Significantly improved survival of SCLC with immunochemotherapy

FDA granted accelerated approval to nivolumab and pembrolizumab as third-line treatment for ES-SCLC in 2018 and 2019, respectively, which has ushered an era of immunotherapy for SCLC. Nevertheless, both approvals were successively withdrawn in 2020 and 2021.^[12,13]^ To the contrary, in March 2019 and March 2020, FDA approved atezolizumab and durvalumab, respectively, in combination with chemotherapy as first-line treatment for patients with ES-SCLC, based on the enthusiastic reports from atezolizumab (IMpower133^[22]^) or durvalumab (CASPIAN^[23]^) in the first-line treatment of ES-SCLC. This has updated the treatment landscape of SCLC from an over-30-year stagnation.^[24]^ Sehhoon Park et al^[14]^ reported the results from a single-arm phase II study in which LS-SCLC patients received durvalumab with concurrent chemoradiotherapy followed by durvalumab consolidation therapy. In this study, patients’ median progression-free survival (PFS) reached 14.4 (95% confidence interval: 10.3-NA) months, and 24-month PFS rate reached 42.0%, indicating the efficacy of durvalumab with concurrent chemoradiotherapy for LS-SCLC.^[14]^ In September 2022, Cheng et al^[15]^ reported the results from an international, double-blind, phase 3 randomized clinical trial (ASTRUM-005) in which ES-SCLC patients received either serplulimab or placebo with chemotherapy. In the study, the serplulimab group achieved both longer median overall survival (13.3 vs 10.9 months) and longer PFS (5.7 vs 4.3 months) than the placebo group, supporting the use of serplulimab plus chemotherapy as first-line treatment for previously untreated ES-SCLC.^[15]^

There have been no clinical trials or case reports published on the backline use of serplulimab for the treatment of SCLC, neither limited-stage or extensive-stage. Yet, in our SCLC with PCS case, the second-line immunochemotherapy consisting of serplulimab with irinotecan-based chemotherapy still presented a satisfactory efficacy.

### 3.2. Efficacy of immunochemotherapy in SCLC complicated with PCS

Although immunochemotherapy has provided generally improved survival for SCLC patients, it’s a little complicated when it comes to SCLC with PCS. On the one hand, some reports have documented the hypercortisolism or PCS induced by immune checkpoint inhibitors (ICI) (combination therapy of ipilimumab and nivolumab in melanoma or monoclonal immunotherapy with pembrolizumab in non-small cell lung cancer).^[25–27]^ On the other hand, glucocorticoids have been universally recommended for ICI-induced adverse effects.^[28–31]^ Nevertheless, previous clinical studies have suggested that the involvement of exogenous glucocorticoid may negatively affect the antitumor efficacy of immunotherapy.^[16–18,32–35]^ Moreover, a latest single-center retrospective study suggested that elevated baseline endogenous glucocorticoid could comprehensively undermine the response to immunotherapy in real-world cancer patients.^[36]^ Furthermore, preclinical studies have also indicated that the activation or signaling of glucocorticoid receptor was associated with immunosuppression, immune evasion, or diminished responses to immunotherapy.^[37–40]^

Ultimately, glucocorticoid exposure may affect the efficacy of immunotherapy and no immunochemotherapy mentioned in past studies on the treatment of SCLC with PCS. Yet, in our SCLC with PCS case, with appropriate control of hypercortisolism, the immunochemotherapy consisting of ICI with platinum-based chemotherapy still presented a satisfactory efficacy.

### 3.3. Lessons from retrospective studies and the current case

The 6 previous retrospective studies have shown the management of SCLC complicated with PCS was dominated by chemoradiotherapy combined with drugs that inhibit or block cortisol synthesis in addition to surgery.^[5–10]^ Unfortunately, the survival status was not so promising. All median survivals were <7 months and a significant proportion of patients died of infections or complications from infections^[5–10]^ (Table 1). In our case, although the patient achieved a long survival of 20 months, as in previous studies, he suffered repeated severe infections that led to interruptions of antitumor treatment. To complicate matters further, the hypercortisolism from PCS could lead to immunosuppression in addition to the immunodeficiency caused by myelosuppression from antitumor therapies.^[41,42]^ Therefore, the management of hypercortisolism, the improvement of myelosuppression, and the prophylaxis against infection are crucial for the continuation of immunochemotherapy and the holistic management of SCLC with PCS patients.

**Table 1:** The 6 retrospective researches on the management of SCLC complicated with PCS.

Author	Year	N	Age	Gender (M/F)	Treatment	Median survival	Cause of death
Dimopoulos et al^[5]^	1992	11	62 (49–65)	3/8	1. Chemotherapy 2. Metyrapone	12 (2–45) d	1. Infection (73%) 2. Cardiac complications 3. Respiratory complications 4. Miscellaneous, not know
Shepherd et al^[7]^	1992	23	60 (43–77)	17/6	1. Chemotherapy 2. Ketoconazole 3. Aminoglutethimide	6.23 (0–20) mo	1. Cancer 2. Infection 3. Pneumonia 4. Other causes
Delisle et al^[9]^	1993	14	62 (36–67)	7/7	1. Chemotherapy 2. Radiotherapy	5.5 (0.75–22) mo	1. Cancer (79%) 2. Infection (21%)
Winquist et al^[8]^	1995	10	58.5 (44–71)	8/2	1. Chemotherapy 2. Ketoconazole	/	1. Cancer 2. Hormonal disorder
Ejaz et al^[10]^	2011	9	/	/	1. Surgery 2. Ketoconazole 3. Metyrapone	/	1. Infection 2. Hyperglycemia 3. Venous thromboembolism
Nagy-Mignotte et al^[6]^	2014	23	62 (29–84)	16/7	1. Chemotherapy 2. Radiotherapy 3. Surgery	6.6 mo (95% CI: 3.2–11.4)	1. Cancer (81.8%) 2. Infection (45.5%) 3. Cardiac complications (9.1%)

CI = confidence interval, F = female, M = male, N = number of patients, PCS = paraneoplastic Cushing syndrome, SCLC = small cell lung cancer.

The current case has its limitations. On the one hand, the case has preliminarily demonstrated the efficacy of immunochemotherapy in the management of SCLC complicated with PCS and the use of serplulimab with irinotecan-based chemotherapy also indicated a satisfactory efficacy as the second-line treatment for ES-SCLC. But the case is only an individual report, its evidence grade is limited and further exploration and validation in a large sample study is necessary. On the other hand, the current report primarily focused on the immunochemotherapy for the primary tumor, but it lacks detailed description or discussion on the treatment of the PCS.

## 4. Conclusion

Here we have presented a rare SCLC with PCS patient who received immunochemotherapy with first-line durvalumab followed by second-line serplulimab, sequentially. From January 2022 to September 2023, the patient achieved a long survival of 20 months. Our case has preliminarily demonstrated the efficacy of immunochemotherapy in the management of SCLC complicated with PCS. And the use of serplulimab with irinotecan-based chemotherapy also indicated its satisfactory efficacy as a second-line treatment for ES-SCLC. The management of hypercortisolism, the improvement of myelosuppression, and the prophylaxis against infection are 3 hinges for the continuation of immunochemotherapy and the holistic management of SCLC with PCS patients. Initial efficacy has been demonstrated, further exploration and validation in a large sample study are awaited.

## Acknowledgments

The authors would like to express their appreciation to the patient and his family.

## Author contributions

**Conceptualization:** Ling Yu, Lizhu Lin.

**Data curation:** Ling Yu, Yanlong Li, Caiyu Li, Yuanliang Li, Lizhu Lin.

**Formal analysis:** Ling Yu, Yanlong Li, Caiyu Li, Xiangjun Qi.

**Investigation:** Ling Yu, Yanlong Li, Hanrui Chen, Lizhu Lin.

**Writing—original draft:** Ling Yu, Yanlong Li, Caiyu Li, Xiangjun Qi, Yeding Lin, Yuanliang Li, Hanrui Chen, Lizhu Lin.

**Writing—review & editing:** Ling Yu, Yanlong Li, Caiyu Li, Xiangjun Qi, Yeding Lin, Yuanliang Li, Hanrui Chen, Lizhu Lin.

**Methodology:** Yanlong Li.

**Software:** Yanlong Li.

**Validation:** Yanlong Li, Hanrui Chen.

**Visualization:** Yanlong Li, Caiyu Li, Xiangjun Qi, Yeding Lin.

**Project administration:** Hanrui Chen, Lizhu Lin.

**Funding acquisition:** Lizhu Lin.

**Resources:** Lizhu Lin.

**Supervision:** Lizhu Lin.
